# Impact of European Association of Urology Combination and Platinum Eligibility on Real‐World Treatment Sequences and Survival in Japanese Patients With Metastatic Urothelial Carcinoma

**DOI:** 10.1111/iju.70403

**Published:** 2026-05-07

**Authors:** Takashi Kawahara, Yoshiyuki Nagumo, Kozaburo Tanuma, Kazuki Hamada, Akane Yamaguchi, Satoshi Nitta, Masanobu Shiga, Atsushi Ikeda, Akio Hoshi, Shuya Kandori, Hiroyuki Nishiyama

**Affiliations:** ^1^ Department of Urology, Faculty of Medicine University of Tsukuba Tsukuba Japan

**Keywords:** combination eligibility, metastatic urothelial carcinoma, platinum eligibility real‐world data, treatment sequence

## Abstract

**Objectives:**

To evaluate the prevalence of European Association of Urology‐defined combination and platinum eligibility at first‐line treatment initiation, and to assess their impact on conventional treatment sequence and overall survival in a real‐world Japanese cohort.

**Methods:**

We retrospectively analyzed 148 patients with metastatic urothelial carcinoma who initiated systemic chemotherapy between 2018 and 2024 at a single institution in Japan. Cisplatin eligibility was assessed according to the Galsky criteria. Combination therapy eligibility and platinum eligibility were defined according to the European Association of Urology guideline.

**Results:**

Among the 148 patients, 128 (86.5%) were combination‐eligible and 20 (13.5%) ineligible. Among combination‐eligible patients, 70 (54.7%) were cisplatin‐eligible, 47 (36.7%) cisplatin‐ineligible but platinum‐eligible, and 11 (8.6%) platinum‐ineligible. Median overall survival was significantly longer in combination‐eligible patients than in combination‐ineligible patients (25 vs. 16 months, *p* = 0.019, HR 2.46, 95% CI 1.16–5.23). Within the combination‐eligible group, cisplatin‐eligible patients tended to show superior median overall survival (not reached) compared with cisplatin‐ineligible/platinum‐eligible (25 months) and platinum‐ineligible (15 months, *p* = 0.11 and *p* = 0.082, respectively). Only 25.0% (32/128) of combination‐eligible patients received the chemotherapy–immune checkpoint inhibitor–enfortumab vedotin sequence, while 20.0% (4/20) of combination‐ineligible patients also completed this sequence.

**Conclusions:**

European Association of Urology‐defined combination and platinum eligibility were associated with survival outcomes and treatment sequence capability in a real‐world setting. These findings highlight the prognostic value but practical limitations of eligibility criteria, emphasizing the need to develop more practical classification frameworks that ensure broader access to effective therapy.

**Abbreviations**
CI: confidence intervals
EAU: European Association of Urology
ECOG: Eastern Cooperative Oncology Group
eGFR: estimated glomerular filtration rate
EV: enfortumab vedotin
EV + *P*
: enfortumab vedotin plus pembrolizumab
GC + Nivo: gemcitabine/cisplatin plus nivolumab
GFR: glomerular filtration rate
HR: hazard ratio
ICI: immune checkpoint inhibitors
mUC: metastatic urothelial cancer
OS: overall survival
PFS: progression‐free survival

## 1 Introduction

The treatment landscape for metastatic urothelial carcinoma (mUC) has undergone a paradigm shift over the past decade. Historically, platinum‐based chemotherapy was the standard first‐line systemic therapy [1]. However, the introduction of immune checkpoint inhibitors (ICIs) and antibody–drug conjugates, such as enfortumab vedotin (EV), has created new opportunities for prolonged survival and durable disease control [2, 3]. In Japan, pembrolizumab became available as a second‐line therapy in 2018, while both avelumab maintenance and EV were introduced into clinical practice in 2022. In 2024, first‐line combination regimens such as EV plus pembrolizumab (EV + *P*) [4] and gemcitabine/cisplatin plus nivolumab (GC + Nivo) [3] have shown superior efficacy compared with that of chemotherapy alone. For patients who progress after platinum‐based chemotherapy, pembrolizumab remains a key second‐line standard of care, as demonstrated in the KEYNOTE‐045 trial [5], while EV has shown an overall survival (OS) benefit in the EV‐301 trial [2]. The JAVELIN Bladder 100 trial further established avelumab maintenance therapy as a standard option for patients who achieved disease control after first‐line chemotherapy [6].

Nevertheless, some patients are ineligible for these therapies because of comorbidities, organ dysfunction, or poor performance status (PS). The European Association of Urology (EAU) guidelines outline criteria for identifying patients potentially suitable for combination regimens, including those with an Eastern Cooperative Oncology Group (ECOG) PS of 0–2, a glomerular filtration rate (GFR) > 30 mL/min, and adequate organ function [7]. These criteria have been integrated into clinical guidelines; however, their prognostic significance in real‐world settings remains insufficiently evaluated. Platinum eligibility, primarily determined by renal function and PS, continues to guide treatment decisions. Cisplatin‐ineligible patients may still receive carboplatin‐based chemotherapy, whereas those ineligible for any platinum agent often begin treatment with ICIs. However, the real‐world impact of platinum ineligibility on long‐term outcomes and treatment transitions in clinical practice remains uncertain [8].

In light of emerging combination therapies such as EV + *P* and GC + Nivo, evaluating past treatment patterns and survival according to EAU‐defined combination and platinum eligibility is important for informing future treatment strategies. We therefore investigated the prevalence and clinical implications of EAU‐defined combination and platinum eligibilities in a Japanese mUC cohort treated between 2018 and 2024. In addition, to compare the prognosis of mUC before and after the introduction of avelumab and EV in 2022, we also compared treatment sequence according to the treatment initiation period: 2018–2021 (pre‐avelumab/EV era) and 2022–24 (post‐avelumab/EV era). We hypothesized that EAU‐defined combination eligibility and platinum eligibility would show substantial discordance in real‐world Japanese practice, thereby influencing treatment selection and clinical outcomes.

## 2 Methods

### 2.1 Study Design and Population

In this retrospective, single‐institution cohort study, we included patients diagnosed with mUC who initiated systemic chemotherapy at our institution between January 2018 and December 2024. In Japan, pembrolizumab became available as a second‐line therapy in 2018, while both avelumab maintenance and EV were introduced into clinical practice in 2022. To assess the impact of the introduction of new therapeutic agents, patients were stratified into two groups according to the treatment initiation period: 2018–2021 (pre‐avelumab/EV era) and 2022 (post‐avelumab/EV era).

### 2.2 Data Collection

Clinical data were extracted from electronic medical records and reviewed retrospectively. Collected variables included demographic characteristics, PS, comorbidities, renal function, cisplatin, platinum eligibility, and EAU‐defined combination therapy eligibility. Each eligibility definition was summarized in Table S1. Cisplatin eligibility was assessed according to the Galsky criteria; patients were considered eligible if they had none of the following: ECOG PS ≥ 2, creatinine clearance < 60 mL/min, grade ≥ 2 hearing loss, grade ≥ 2 peripheral neuropathy, or New York Heart Association class III/IV heart failure. Platinum eligibility was defined using the EAU guidelines; patients were considered eligible if they had none of the following: GFR < 30 mL/min, ECOG PS > 2, ECOG PS = 2 with a GFR < 60 mL/min, or comorbidities of grade > 2. Combination therapy eligibility was defined according to the EAU guidelines; patients were considered eligible if they had an ECOG PS of 0–2, GFR > 30 mL/min, and adequate organ function. Renal function was primarily assessed using the estimated GFR (eGFR). In cases where impaired renal function was suspected based on eGFR values, 24‐h creatinine clearance was additionally measured to more accurately evaluate renal function. Treatment sequences, including first‐, second‐, and third‐line regimens, were evaluated. In this study, avelumab maintenance therapy was categorized as second‐line treatment because it was administered after completion of first‐line chemotherapy in patients without disease progression.

### 2.3 Endpoints

The primary endpoint was the prevalence and survival outcomes of EAU‐defined combination and platinum eligibility at first‐line treatment initiation. Additionally, we examined treatment sequence patterns, transitions to second‐ and third‐line therapies stratified by eligibility classifications and treatment period (2018–2021 (pre‐avelumab/EV era) and 2022–2024 (post‐avelumab/EV era)). For the survival analyses, the data cutoff date was May 31, 2025.

### 2.4 Statistical Analysis

Descriptive statistics were used to summarize the patient characteristics and treatment patterns. Categorical variables are presented as frequencies and percentages, and continuous variables as medians and ranges. OS was estimated using the Kaplan–Meier method and compared using the log‐rank test. Stratified analyses were conducted according to treatment period, platinum eligibility, and EAU combination eligibility. All statistical analyses and survival curves were generated using GraphPad Prism (version 10.0; GraphPad Software, San Diego, CA, USA). Statistical significance was set at a two‐sided *p* < 0.05. Sankey diagrams were created using SankeyMATIC (https://sankeymatic.com).

## 3 Results

### 3.1 Patient Characteristics

A total of 148 patients with mUC who initiated systemic chemotherapy between 2018 and 2024 were included. Table 1 summarizes patient characteristics. The median age was 73 years (range, 36–86), and 72% were male. Most patients had an ECOG PS of 0 or 1. Primary tumor locations included the upper urinary tract in 49 patients (33.1%), in the lower tract in 84 (56.8%), and in both sites in 15 (10.1%). Table 2 summarizes the cross‐distribution of eligibility according to the criteria defined in Table S1. Among 70 cisplatin‐eligible patients, all were EAU combination eligible. Among the 54 patients who were cisplatin ineligible but platinum eligible, 47 were EAU combination eligible and 7 were ineligible. Among the 24 platinum‐ineligible group, 11 were EAU combination eligible and 13 were ineligible. Detailed distributions of the EAU‐defined combination‐ineligible factors and their combinations are presented in Table 3. In terms of treatment period, 70 (47.3%) patients were treated in the pre‐avelumab/EV era, and 78 (52.7%) patients were treated in the post‐avelumab/EV era. Table 4 summarizes patient characteristics according to the treatment period, and there were no significant differences between the two groups.

**TABLE 1:** Patient characteristics.

		Number (%)
Median age (range)		73 (36–86)
Sex	Male	107 (72.3)
	Female	41 (27.7)
Tumor location	Upper	49 (33.1)
	Lower	84 (56.8)
	Both	15 (10.1)
Performance status	0	106 (71.6)
	1	30 (20.3)
	2	12 (8.1)
Treatment period	2018–2021 (**pre‐avelumab/EV era**)	70 (47.3)
	2022–2024 (**post‐avelumab/EV era**)	78 (52.7)
Cisplatin eligibility	Eligible	70 (47.3)
	Ineligible	78 (52.7)
Platinum eligibility	Eligible	124 (83.8)
	Ineligible	24 (16.2)
EAU combination eligibility	Eligible	128 (86.5)
	Ineligible	20 (13.5)

**TABLE 2:** Distribution of patients by EAU combination eligibility and cisplatin/platinum eligibility.

	EAU combination eligible	EAU combination ineligible
Cisplatin eligible	70	0
Cisplatin ineligible/platinum eligible	47	7
Platinum ineligible	11	13

**TABLE 3:** Detailed information of EAU‐defined combination ineligible patients.

Factors	*n* (%)
Organ dysfunction	9^a^ (45.0)
Ileus	2 (10.0)
Autoimmune diseases	5 (25.0)
Interstitial lung disease	2 (10.0)
Renal impairment GFR < 30	12^a^ (60.0)
PS ≥ 3	0

^a^ One patient had two factors.

**TABLE 4:** Patient characteristics according to the treatment period.

		Pre‐avelumab/EV era (2018–21) *n* = 70	Post‐avelumab/EV era (2022–24) *n* = 78	*p*
Median age (range)		73 (36–86)	74 (47–84)	0.7613
Sex	Male	47	60	0.1844
	Female	23	18	
Tumor location	Upper	22	27	0.5742
	Lower	39	45	
	Both	9	6	
Performance status	0	51	55	0.5901
	1	15	15	
	2	4	8	
Cisplatin eligibility	Eligible	30	40	0.3054
	Ineligible	40	38	
Platinum eligibility	Eligible	56	68	0.2368
	Ineligible	14	10	
EAU‐defined combination eligibility	Eligible	59	69	0.4581
	Ineligible	11	9	

### 3.2 Reasons for Cisplatin and Platinum Ineligible

Among the cisplatin‐ineligible patients, the most common reason was impaired renal function (88.4%, 69/78), followed by poor PS (15.4%, 12/78), preexisting neuropathy (9.0%, 7/78), and heart failure (1.3%, 1/78). Seven patients met two of these criteria, and one met three of these criteria. For platinum ineligibility, the main reasons were severe renal impairment (50.0%, 12/24), grade ≥ 3 comorbidities (29.2%, 7/24), and PS 2 combined with moderate renal impairment (GFR 30–60 mL/min, 20.8%, 5/24). One patient met two of these ineligibility criteria.

### 3.3 Treatment Sequences, Patterns, and Prognosis by Cisplatin and Platinum Eligibility

**FIGURE 1:**
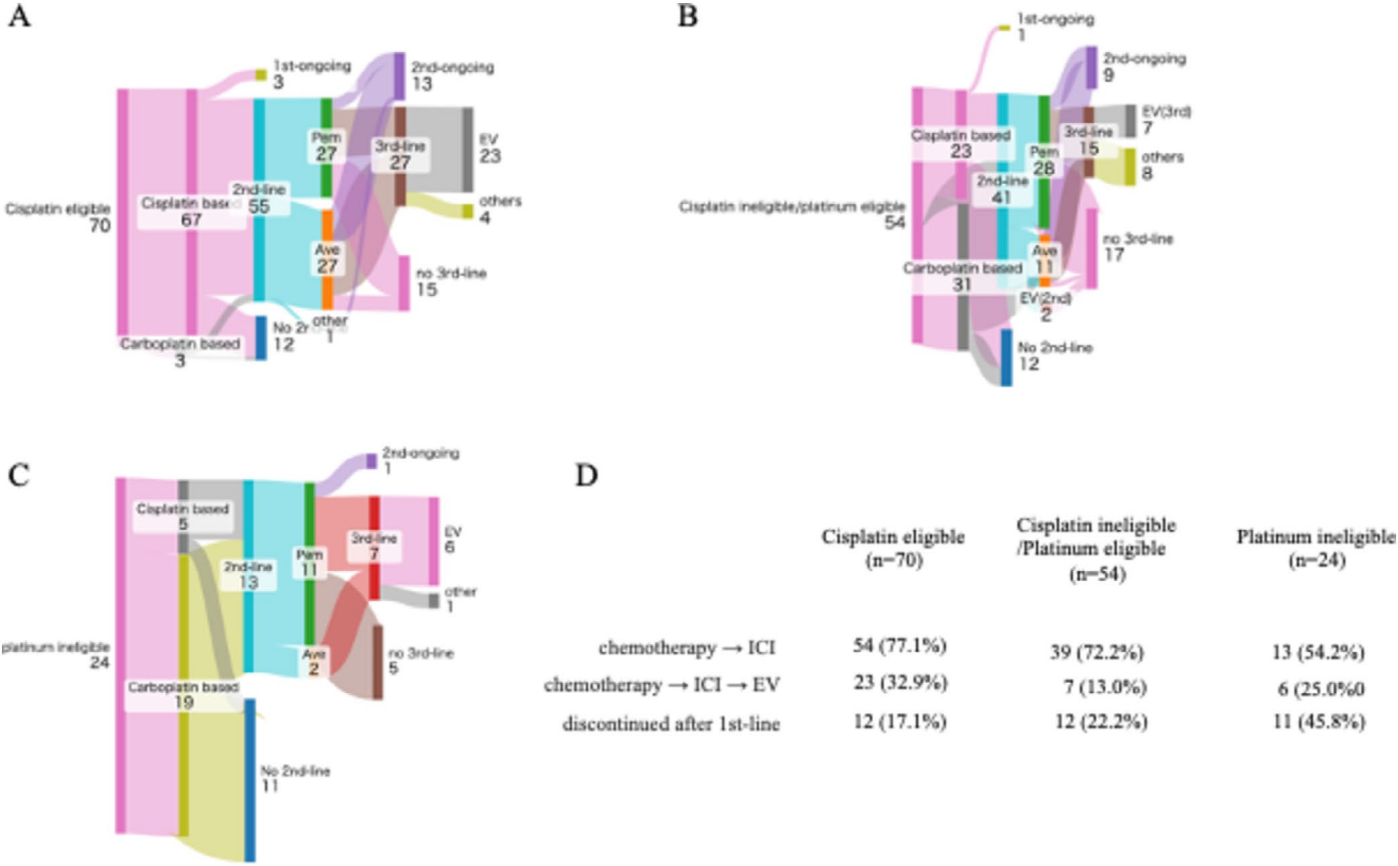
Sankey diagram of treatment sequences patterns in cisplatin‐eligible patients (A), cisplatin‐ineligible and platinum eligible patients (B), and platinum ineligible patients (C). Summary of treatment sequences (D).

**FIGURE 2:**
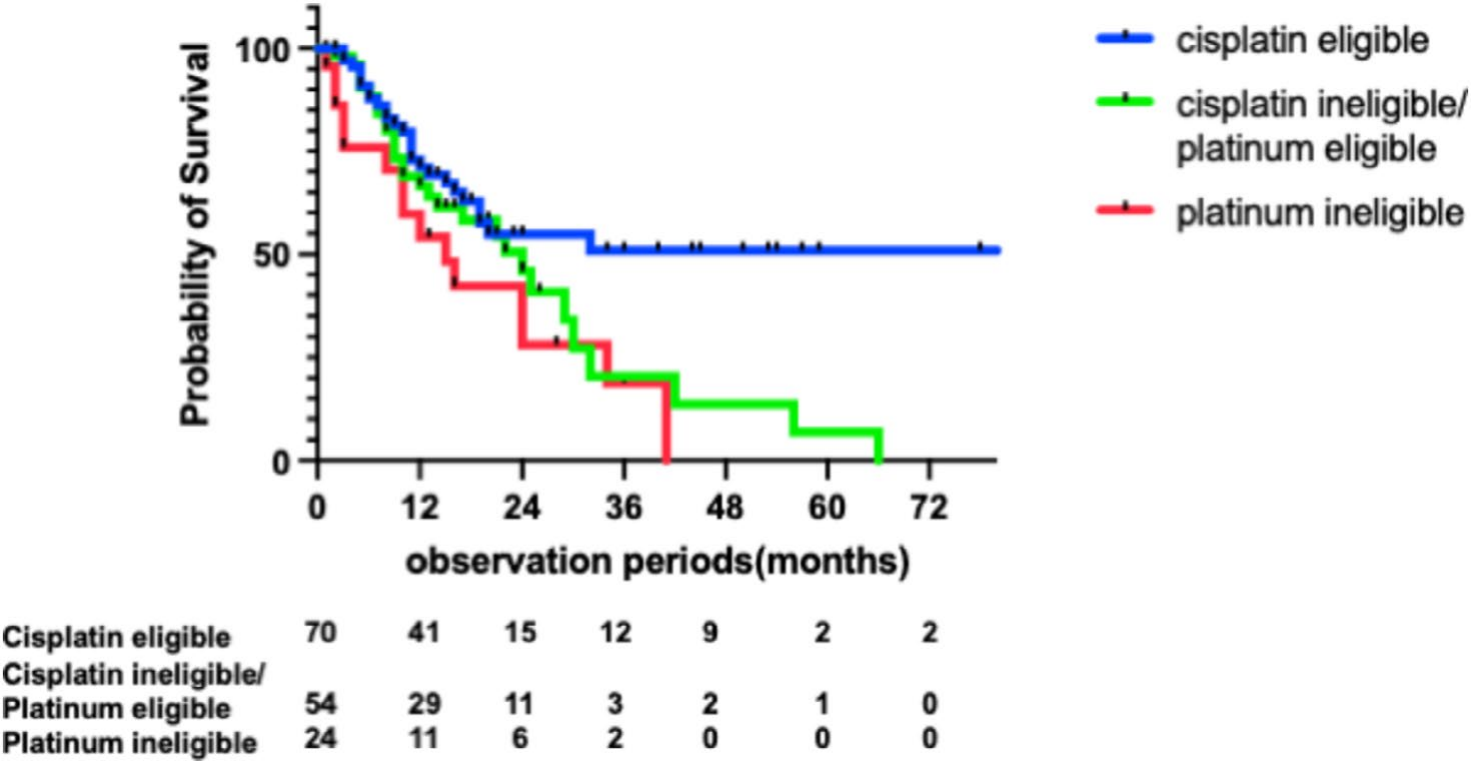
Overall survival (OS) by the combination of cisplatin and platinum eligibility.

In the cisplatin‐eligible group (*n* = 70), most patients (95.7%, 67/70) received cisplatin‐based first‐line chemotherapy. Of these, 55 (78.6%) transitioned to second‐line treatment, mainly with pembrolizumab (49.1%, 27/55) or avelumab (49.1%, 27/55) (Figure 1A). A total of 27 patients (38.6%, 27/70) subsequently received third‐line therapy. On the other hand, in the cisplatin‐ineligible/platinum‐eligible group (*n* = 54), thirty‐one (57.4%) patients received carboplatin‐based chemotherapy and 23 (42.6%) received cisplatin‐based regimens (Figure 1B). Of these, 41 (75.9%) patients proceeded to second‐line therapy (pembrolizumab, *n* = 28; avelumab, *n* = 11), and 15 (27.8%, 15/54) subsequently received third‐line treatment. In the platinum ineligible group (*n* = 24), most patients (79.2%, 19/24) received carboplatin‐based chemotherapy (Figure 1C). Of these, 13 (54.2%, 13/24) advanced to second‐line therapy, and 7 (29.2%, 7/24) proceeded to third‐line treatment. Notably, this group showed the highest discontinuation rate after first‐line therapy (*n* = 11, 45.8%). The cisplatin‐eligible patients showed longer median OS (Not reached) compared with cisplatin‐ineligible/platinum‐eligible and platinum‐ineligible (24 months and 15 months, *p* = 0.054 and *p* = 0.0082, respectively) (Figure 2).

### 3.4 Association Between EAU Combination, Cisplatin and Platinum Eligibility: Impact on Treatment Sequences and Prognosis

Among the 128 EAU combination‐eligible patients, 70 (54.7%) were cisplatin‐eligible, 47 (36.7%) were cisplatin‐ineligible/platinum‐eligible, and 11 (8.6%) were platinum‐ineligible. In contrast, among the 20 EAU combination‐ineligible patients, none were cisplatin‐eligible; seven (35.0%) were cisplatin‐ineligible/platinum‐eligible, and 13 (65.0%) were platinum‐ineligible.

**FIGURE 3:**
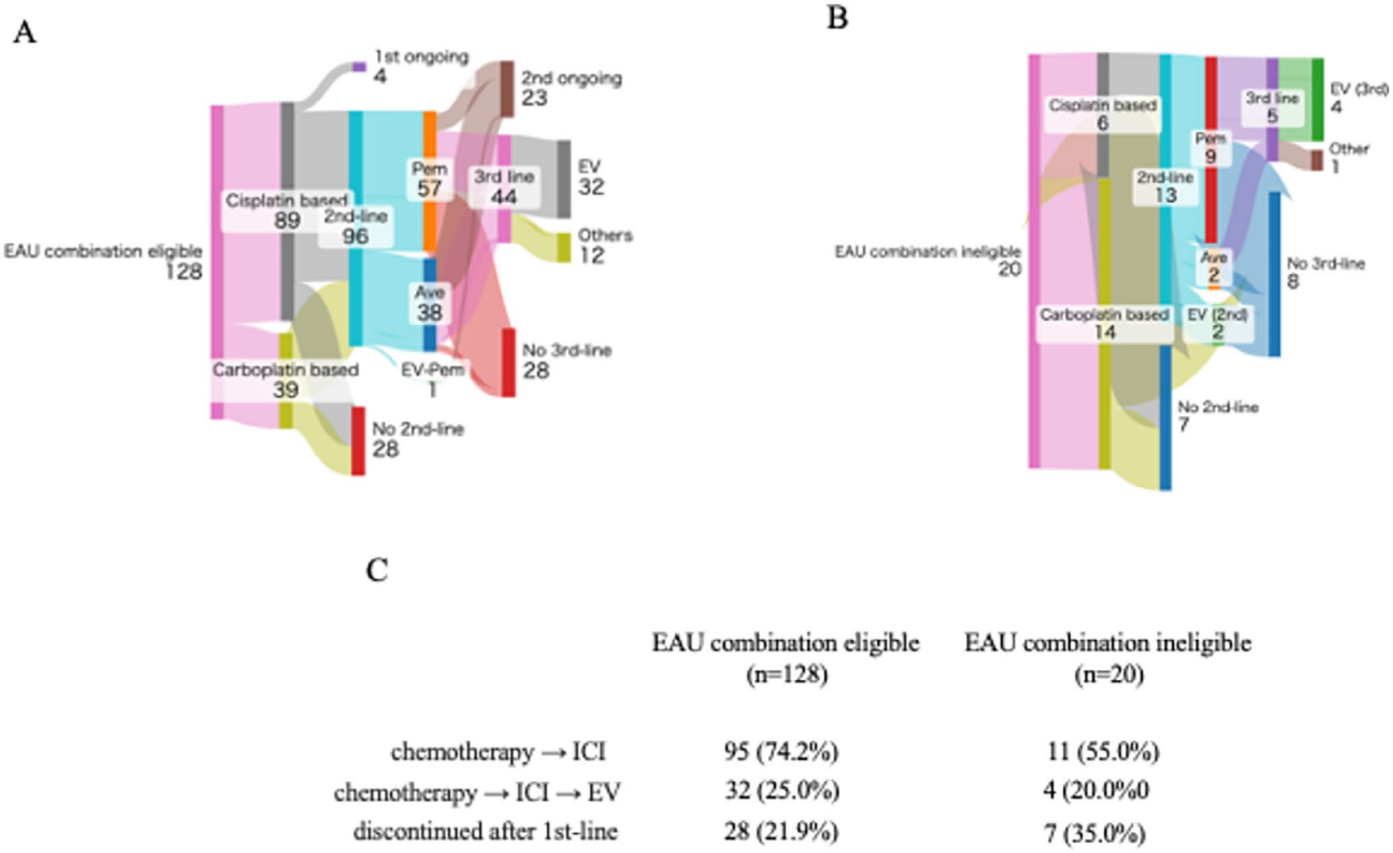
Sankey diagram of treatment sequences patterns in EAU combination eligible patients (A) and ineligible patients (B). Summary of treatment sequences (C).

**FIGURE 4:**
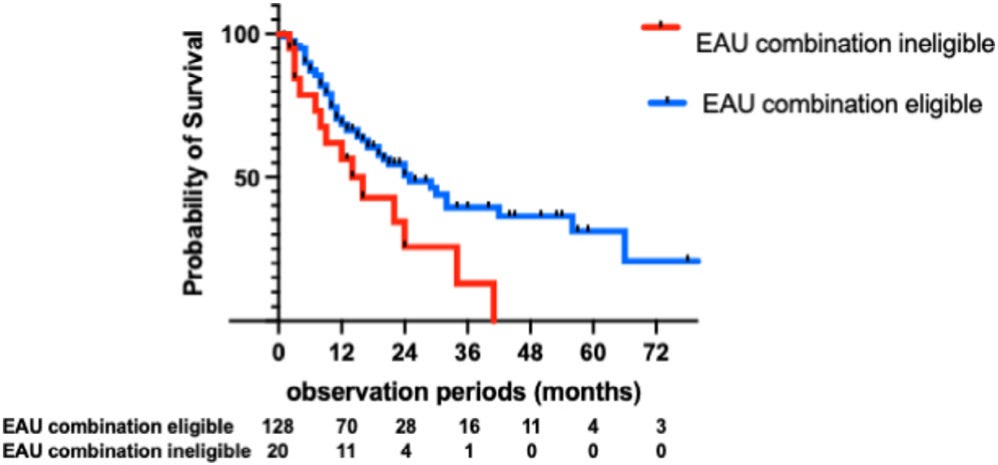
OS stratified by EAU combination eligibility status.

Among the 128 EAU combination‐eligible patients, 28 (21.9%) received chemotherapy alone without transitioning to ICI therapy, while the remaining 95 patients (74.2%) underwent sequential treatment with chemotherapy, followed by ICI (Figure 3A). At the time of data cutoff, 32 patients received EV as a third‐line therapy, and three received EV in the fourth or subsequent lines. Overall, 35 patients (27.3%, 35/128) received sequential treatment consisting of chemotherapy followed by ICI therapy and subsequent EV. As shown in Figure 3B, among the 20 EAU combination‐ineligible patients, seven (35.0%) received chemotherapy alone without transitioning to ICI or EV therapy, 11 (55.0%) underwent sequential treatment with chemotherapy followed by ICI, two (10.0%) received chemotherapy followed by EV as second‐line therapy, and four (20.0%) received sequential therapy consisting of chemotherapy, ICI, and EV. OS was significantly longer in patients classified as EAU combination eligible than in those deemed ineligible (median OS: 25 vs. 16 months, *p =* 0.0191 Hazard Ratio (HR) 2.46, 95% confidence intervals (95% CI) 1.16–5.23) (Figure 4).

### 3.5 Survival Outcomes in EAU Combination Eligible Patients: Impact of Platinum Eligibility, Treatment Sequences, and Treatment Period

**FIGURE 5:**
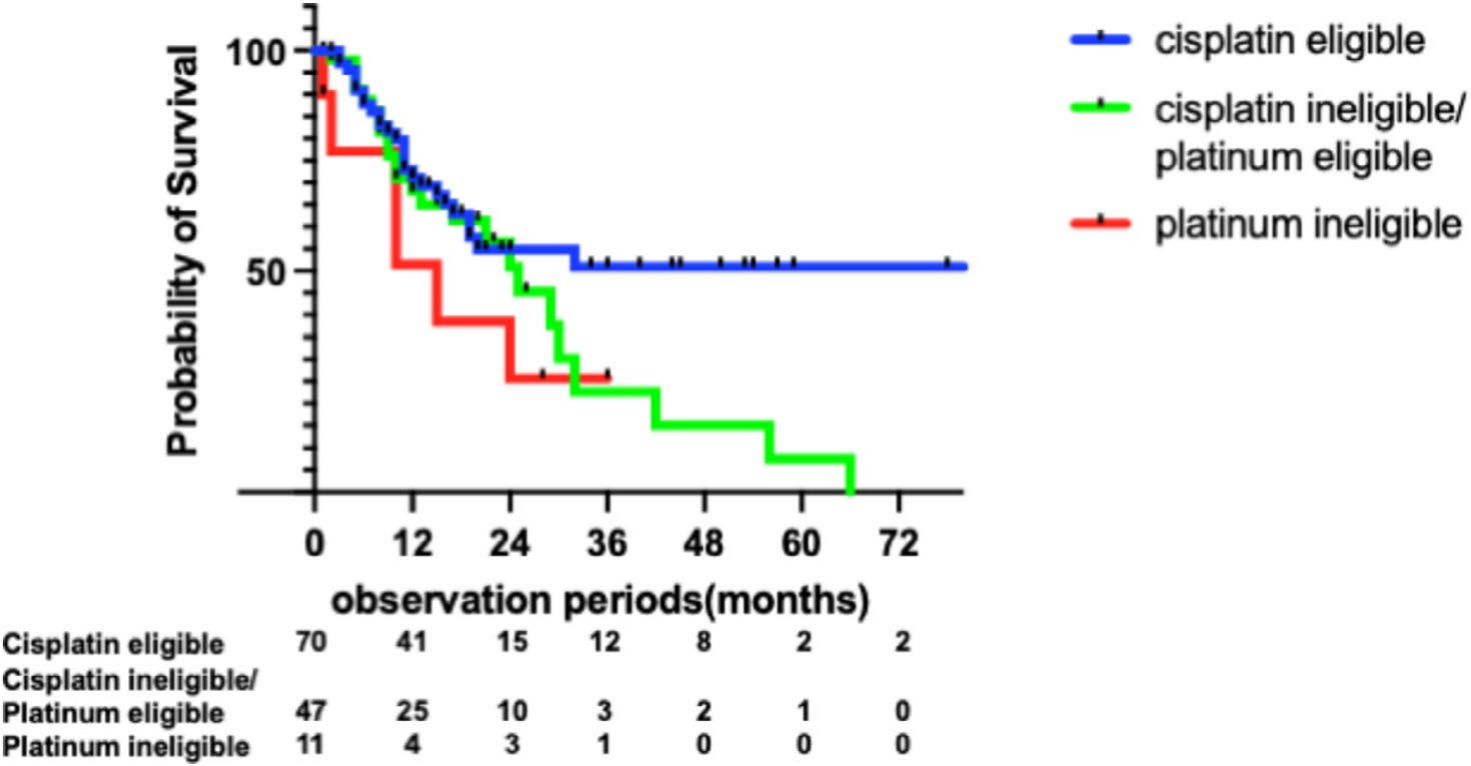
OS by cisplatin and platinum eligibility among EAU combination eligible patients.

**FIGURE 6:**
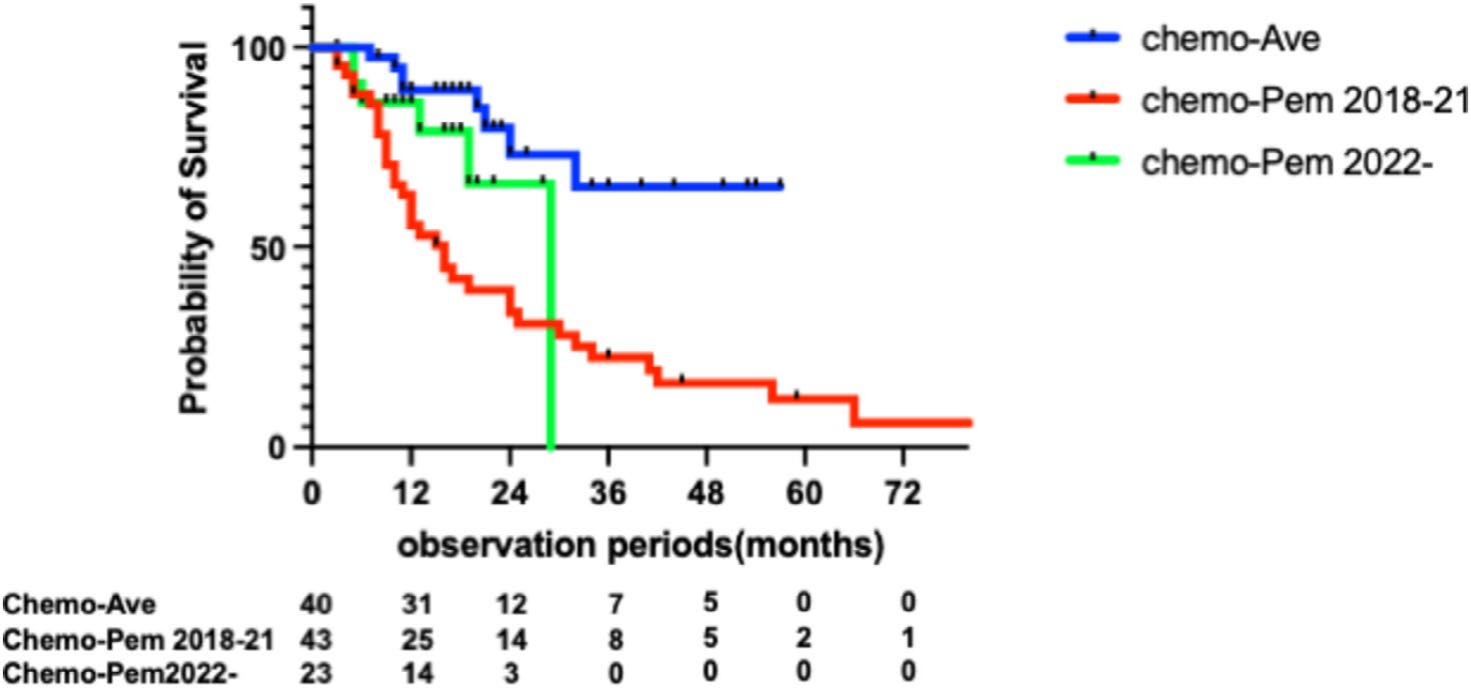
OS according to post‐chemotherapy treatment sequences and treatment period among EAU combination eligible patients.

Within the combination‐eligible group, cisplatin‐eligible patients showed a non‐significant trend toward longer median overall survival (not reached) compared with cisplatin‐ineligible/platinum‐eligible (25 months, *p* = 0.11, HR: 1.61, 95% CI, 0.90–2.89), and also did not show significantly longer survival compared with platinum‐ineligible patients (15 months, *p* = 0.082, HR 2.86, 95% CI: 0.88–9.35) (Figure 5). Notably, among them, patients treated with chemotherapy followed by avelumab maintenance therapy achieved the longest median OS, which was not reached. Conversely, patients who received chemotherapy followed by pembrolizumab had a median OS of 16 months in the pre‐avelumab/EV era, which improved to 29 months in the post‐avelumab/EV era (Figure 6).

## 4 Discussion

The clinical implications of EAU‐defined combination ineligibility remain underexplored. In this study, we focused on the clinical relevance of EAU‐defined ineligibility for combination therapy [7] in patients with mUC. The prognostic significance of EAU‐defined ineligibility had not been specifically evaluated; however, our findings show that 13.5% of the patients met the ineligibility criteria at first‐line treatment initiation, primarily owing to comorbidities such as renal impairment, autoimmune disease, or interstitial lung disease. These patients demonstrated significantly worse OS. Furthermore, EAU combination eligibility was associated with the ability to receive sequential systemic therapies.

Although cisplatin eligibility and the EAU‐defined combination eligibility framework serve as important references for initial treatment decision‐making, our findings suggest that these dichotomous criteria do not fully reflect real‐world treatment feasibility. While most EAU combination eligible patients underwent treatment with chemotherapy followed by ICI, only 27.3% received the full sequence of chemotherapy, ICI, and EV. Interestingly, 20.0% of EAU combination ineligible patients also completed this treatment sequence. Likewise, several patients classified as EAU combination ineligible successfully received sequential systemic therapies, indicating that ineligibility does not uniformly preclude progression to later treatment lines. Collectively, these observations highlight the limitations of rigid, binary eligibility frameworks when applied to heterogeneous, comorbid, and aging populations. Future research should focus on refining these criteria and establishing more flexible, clinically meaningful algorithms that incorporate comorbidities, organ function, treatment tolerance, and patient preferences to better guide safe and effective therapeutic planning.

Recent Japanese data suggest that many patients with mUC receive first‐line platinum‐based chemotherapy followed by ICIs or EV, and that such treatment sequences contribute to improved survival [9, 10]. However, in real‐world practice, not all patients can complete these optimal sequences. Kita et al. [9] reported that baseline factors such as elevated neutrophil‐to‐lymphocyte ratio, low body mass index, and poor PS were significant barriers to receiving later‐line ICIs or EV and were associated with worse survival. Similarly, Urabe et al. [10] observed that only a limited proportion of patients transitioned from chemotherapy to avelumab maintenance or EV, often hindered by older age, poor PS, or elevated C‐reactive protein levels. In our study, we uniquely evaluated treatment feasibility using EAU‐defined combination eligibility, a framework conceptually distinct from these previously reported risk factors. As a referral institution, our center may receive patients with strong treatment motivation, which could influence treatment decisions and contribute to selection bias. Consequently, EAU combination eligibility alone did not fully determine sequential therapy feasibility in our cohort. These findings underscore the importance of interpreting eligibility criteria within the institutional context and considering additional baseline clinical factors when planning treatment strategies for patients with mUC.

Platinum eligibility also strongly influenced treatment sequences and overall prognosis in patients with mUC. In our cohort, 47.3% were cisplatin‐eligible, 36.5% were cisplatin‐ineligible but platinum‐eligible, and 16.2% were platinum‐ineligible. These classifications corresponded to distinct treatment pathways and survival outcomes. Cisplatin‐eligible patients had the highest transition rates to second‐ (78.6%) and third‐line therapy (38.6%), with an unreached median OS. In contrast, platinum‐ineligible patients had more limited access to subsequent therapy (54.2% received second‐ and 29.2% third‐line treatment) and a significantly shorter OS (median: 15 months). These findings align with those of previous studies, supporting cisplatin eligibility as a surrogate marker for preserved PS and organ function, both of which influence prognosis and treatment tolerance [8]. Notably, however, a substantial proportion of platinum‐ineligible patients received platinum agents—including carboplatin—in real‐world practice, highlighting a discrepancy between strict eligibility definitions and practical treatment feasibility. Moreover, because a subset of platinum‐ineligible patients also received cisplatin‐based therapy, the pure effect of carboplatin use on overall survival cannot be isolated in this study. Although platinum‐ineligible patients showed poorer survival, this pattern is likely driven by underlying frailty and comorbidities rather than the choice of platinum compound alone. Therefore, we refrained from causal interpretation and limited our explanation to a descriptive interpretation of the observed trend.

Further analysis stratified by post‐chemotherapy sequences revealed that patients receiving avelumab maintenance therapy achieved the most favorable outcomes, with a median OS not reached. Importantly, survival outcomes improved significantly in the post‐2022 era, when EV became available in Japan. The JAVELIN Bladder 100 trial, the findings of which established the benefit of avelumab maintenance after platinum‐based chemotherapy [6], and the J‐AVENUE study, a Japanese real‐world analysis, confirmed its feasibility and tolerability [11]. Additionally, in a recent multicenter Japanese study, patients receiving EV after avelumab maintenance achieved significantly longer OS compared with those treated with EV following pembrolizumab (median OS: 16.0 vs. 10.2 months, *p =* 0.019), despite no significant difference in progression‐free survival (median PFS: 6.4 vs. 4.2 months, *p =* 0.184) [12]. Notably, among patients treated with pembrolizumab following chemotherapy, the median survival appeared to have improved from 16 months in the 2018–2021 period to 29 months in the 2022–2024 period, potentially reflecting increased EV availability.

This study has some limitations. First, it was a single‐center, retrospective analysis, which may have introduced selection bias and limited generalizability. Data were collected consecutively; however, patient characteristics and treatment patterns may reflect institutional preferences or referral biases. Future multicenter validation or integration with large‐scale registries, such as the Urothelial Cancer Registry, is expected to overcome these limitations by increasing sample size and enhancing external validity. Second, the assessment of platinum and EV therapy eligibility was based on a retrospective chart review; certain clinical parameters, such as the presence of comorbidities, functional decline, and aging, were subject to clinical judgment. Third, the modest sample size, particularly in the EAU combination ineligible and platinum‐ineligible subgroups, may have reduced the statistical power and limited the ability to detect small differences in survival outcomes. Fourth, we did not include emerging combination therapies (such as EV + *P* and GC + Nivo), as these regimens were not widely adopted in Japan during the study period. Therefore, the results may not fully reflect the evolving treatment landscape. Finally, although we stratified the patients by treatment period (pre‐2022 vs. post‐2022) to account for avelumab and EV availability, unmeasured confounders related to changes in supportive care, patient selection, or broader practice patterns over time may have influenced the outcomes. Despite these limitations, our study offers valuable insights into real‐world eligibility, treatment sequences, and survival outcomes in a contemporary Japanese cohort of patients with mUC.

In conclusion, this study showed that EAU‐defined combination and platinum eligibility were associated with treatment sequence capability and survival outcomes in a real‐world, single‐institutional Japanese mUC cohort. Our findings suggest that the therapeutic landscape post‐2022, marked by avelumab and EV availability, offers enhanced opportunities for sequential therapy and survival benefit, particularly in patients initially eligible for platinum‐based treatment. Further studies are warranted to validate these findings and develop more practical classification frameworks that ensure broader access to effective therapy for patients with mUC.

## Author Contributions

**Takashi Kawahara:** conceptualization, methodology, project administration, writing – original draft preparation. **Yoshiyuki Nagumo:** methodology, writing – original draft preparation. **Kozaburo Tanuma:** data curation, investigation. **Kazuki Hamada:** data curation, investigation. **Akane Yamaguchi:** formal analysis. **Satoshi Nitta:** investigation. **Masanobu Shiga:** investigation. **Atsushi Ikeda:** writing – review and editing. **Akio Hoshi:** supervision, writing – review and editing. **Shuya Kandori:** project administration, writing – review and editing. **Hiroyuki Nishiyama:** supervision, writing – review and editing.

## Disclosure

Hiroyuki Nishiyama is an Editorial Board member of International Journal of Urology and a co‐author of this article. To minimize bias, they were excluded from all editorial decision‐making related to the acceptance of this article for publication. The other authors declare no conflict of interest.

## Ethics Statement

This study was conducted in accordance with the principles of the Declaration of Helsinki and was approved by the Internal Review Board of the Tsukuba University Hospital (approval number: H29‐030). Informed consent was obtained by the opt‐out method through the hospital website. Patients who opted out of the study were excluded.

## Consent

The authors have nothing to report.

## Conflicts of Interest

The authors declare no conflicts of interest.

## Supporting information


**Table S1:** Definitions of each eligibility categories.

## Data Availability

The datasets generated and analyzed during the current study are not publicly available due to patient privacy concerns. However, they are available from the corresponding author on reasonable request and with appropriate institutional approval.
